# 'SpineCor' apical vertebral rotation measuring tool

**DOI:** 10.1186/1748-7161-8-S2-O19

**Published:** 2013-09-18

**Authors:** Alison Murray, Tim Cook

**Affiliations:** 1 SpineCorporation Ltd, Montreal, QC, Canada

## Background

The measurement of Apical Vertebral Rotation (AVR) is vital to the surgical and conservative treatment of idiopathic scoliosis. Several measuring methods are used to assess the AVR on plain radiographs, including the visual Nash and Moe, Perdriolle’s torsion meter and Raimondi’s table, all of which have positive and negative aspects[1-3].

## Purpose

We developed an AVR image scale of 0-55° rotation, pre-determined, to assess its ease of use as a visual method when comparing the mean inter- and intra-reliability against Perdriolle’s and Raimondi’s scales.

## Method

A human lumbar vertebra (L4) set on a rotating device, and a series of X rays were taken, at 1 degree increments, from 0-55° clockwise rotation. These images were flipped to produce 0-55° counterclockwise rotation. The apical rotations of 39 curves was measured by three different observers (each having more than three years of experience specializing in the field of scoliosis) using the SpineCor AVR scale, Perdriolle’s torsion meter and Raimondi’s table. The measurements were taken twice, with a minimum of one week between each measurement.

## Results

The inter-rater mean difference for the SpineCor AVR scale versus both Perdriolle’s and Raimondi’s was the same (within 0.01°) but this mean difference was greater than the mean difference between Raimondi’s and Perdriolle’s by 0.48° and 0.49° respectively (SpineCor AVR scale versus Raimondi = mean difference of 4.18° SD+/- 2.04°, SpineCor AVR scale v Perdriolle’s = mean difference 4.19° SD +/- 2.05°, Raimondi’s v Perdriolle’s = 3.70° SD +/- 1.92°); the intra-rater mean difference was 1.07° greater with the SpineCor AVR scales than with Perdriolle’s and 0.45° greater than Raimondi’s table (intra-rater mean difference for the three testers with SpineCor images= 2.29° SD +/- 0.44°, with Raimondi’s = 1.84° SD +/- 0.39° and with Perdriolle’s = 1.22° SD +/- 0.44°).

## Conclusions and discussion

The SpineCor AVR scales demonstrated high intra-reliability mean differences with a mean difference of 2.29° SD +/- 0.44° and inter-reliability of 4.18° +/- 2.04° SD and 4.19° +/- 2.05° SD, which does appear to demonstrate favourable intra- and inter-reliability in comparison to both Perdriolle’s and Raimondi’s scales[1,2].
